# Biological sex impacts oxidative stress in skeletal muscle in a porcine heat stress model

**DOI:** 10.1152/ajpregu.00268.2023

**Published:** 2024-05-06

**Authors:** Tori E. Rudolph, Melissa Roths, Alyssa D. Freestone, Sau Qwan Yap, Alyona Michael, Robert P. Rhoads, Sarah H. White-Springer, Lance H. Baumgard, Joshua T. Selsby

**Affiliations:** ^1^Department of Animal Science, Iowa State University, Ames, Iowa, United States; ^2^Department of Vet Diagnostic & Production Animal Med, Iowa State University, Ames, Iowa, United States; ^3^School of Animal Sciences, Virginia Tech, Blacksburg, Virginia, United States; ^4^Department of Animal Science, Texas A&M University and Texas A&M AgriLife Research, College Station, Texas, United States; ^5^Department of Kinesiology and Sport Management, Texas A&M University, College Station, Texas, United States

**Keywords:** calcium, climate change, heat stroke, hyperthermia, pig

## Abstract

Oxidative stress contributes to heat stress (HS)-mediated alterations in skeletal muscle; however, the extent to which biological sex mediates oxidative stress during HS remains unknown. We hypothesized muscle from males would be more resistant to oxidative stress caused by HS than muscle from females. To address this, male and female pigs were housed in thermoneutral conditions (TN; 20.8 ± 1.6°C; 62.0 ± 4.7% relative humidity; *n* = 8/sex) or subjected to HS (39.4 ± 0.6°C; 33.7 ± 6.3% relative humidity) for 1 (HS1; *n* = 8/sex) or 7 days (HS7; *n* = 8/sex) followed by collection of the oxidative portion of the semitendinosus. Although HS increased muscle temperature, by 7 days, muscle from heat-stressed females was cooler than muscle from heat-stressed males (0.3°C; *P* < 0.05). Relative protein abundance of 4-hydroxynonenal (4-HNE)-modified proteins increased in HS1 females compared with TN (*P* = 0.05). Furthermore, malondialdehyde (MDA)-modified proteins and 8-hydroxy-2′-deoxyguanosine (8-OHdG) concentration, a DNA damage marker, was increased in HS7 females compared with TN females (*P* = 0.05). Enzymatic activities of catalase and superoxide dismutase (SOD) remained similar between groups; however, glutathione peroxidase (GPX) activity decreased in HS7 females compared with TN and HS1 females (*P* ≤ 0.03) and HS7 males (*P* = 0.02). Notably, HS increased skeletal muscle Ca^2+^ deposition (*P* = 0.05) and was greater in HS1 females compared with TN females (*P* < 0.05). Heat stress increased sarco(endo)plasmic reticulum Ca^2+^ ATPase (SERCA)2a protein abundance (*P* < 0.01); however, Ca^2+^ ATPase activity remained similar between groups. Overall, despite having lower muscle temperature, muscle from heat-stressed females had increased markers of oxidative stress and calcium deposition than muscle from males following identical environmental exposure.

**NEW & NOTEWORTHY** Heat stress is a global threat to human health and agricultural production. We demonstrated that following 7 days of heat stress, skeletal muscle from females was more susceptible to oxidative stress than muscle from males in a porcine model, despite cooler muscle temperatures. The vulnerability to heat stress-induced oxidative stress in females may be driven, at least in part, by decreased antioxidant capacity and calcium dysregulation.

## INTRODUCTION

With more frequent and severe heat events, heat stress (HS) is a present and mounting concern for public health and animal agriculture (1–4). Heat stress is physiologically distinct from both therapeutic hyperthermia and heat stroke, as the former may enhance muscle growth (5, 6), whereas the latter typically includes central nervous system dysfunction and can result in death (7). Currently, the pathological consequences of HS are poorly defined; a knowledge gap made even more critical as ∼70% of heat-related hospitalizations are due to HS (8, 9). In addition to threatening human health, HS negatively impacts agricultural production (10, 11), particularly as continued genetic selection for rapid growth has increased metabolic heat production, and therefore increased susceptibility to the negative effects of environmental heat (12). Productivity losses accrued following HS events are detrimental to agricultural economics, increase the environmental strain of production, and increasingly threaten global food security in the face of current and accelerating climate change (13, 14).

Oxidative stress and subsequent dysfunction are common sequela of environment-induced HS in skeletal muscle (15–20). This is notably divergent from the reported antioxidant effects of therapeutic hyperthermia (5, 6). Heat stress causes reduced mitochondrial function, mitochondrial remodeling, and alters markers of mitophagy in oxidative skeletal muscle (21–23). Loss of Ca^2+^ homeostatic control is regularly observed during muscle injury and disease (24–27) and may lead to mitochondrial Ca^2+^ sequestration (28, 29) and subsequent oxidative stress following impaired function of sarco(endo)plasmic reticulum Ca^2+^ ATPase (SERCA). Indeed, SERCA function may be impaired due to thermal denaturation (30, 31) and oxidative modification (32), both of which are likely during HS. Importantly, Ca^2+^ dysregulation was causatively linked to mitochondrial dysregulation in a *Caenorhabditis elegans* HS model (33).

Recent data from our laboratory using a porcine heat-stress model suggest that females are more susceptible to skeletal muscle dysfunction than males (34, 35). Specifically, we had consistently identified oxidative stress and dysfunctional autophagy in skeletal muscle from females (15, 17, 18, 22, 36); however, in a recent investigation, males appeared resistant to these HS-induced changes (34). Nevertheless, the extent to which biological sex impacts oxidative stress following HS remains largely unknown. We hypothesized that muscle from males would better preserve redox balance following environment-induced HS than muscle from females.

## MATERIALS AND METHODS

All procedures were reviewed and approved by the Institutional Animal Care and Use Committee at Iowa State University (No. 20-063). A more detailed study design and animal treatments have been previously described (35, 37). Briefly, 3-mo-old crossbred males (castrated male pigs; barrows) and prepubescent females (gilts) [36.8 ± 3.7 kg body wt (BW)] were housed in either thermoneutral (TN) conditions (20.8 ± 1.6°C; 62.0 ± 4.7% relative humidity; *n* = 8/sex) or exposed to constant HS (39.4 ± 0.6°C; 33.7 ± 6.3% relative humidity) for either 1 (HS1; *n* = 8/sex) or 7 days (HS7; *n* = 8/sex). After the environmental challenge, all pigs were euthanized via captive bolt and exsanguination. Immediately following euthanasia, an incision was made to expose the semitendinosus, and a calibrated digital thermometer (Taylor, Model 3519) was inserted into the muscle belly to measure muscle temperature (*T*_m_). The contralateral semitendinosus was excised, and the red (oxidative) portion collected and frozen in liquid nitrogen or fixed in 4% paraformaldehyde for subsequent analyses.

### Protein Extraction and Western Blot

Protein was extracted and Western blot analysis performed as previously described (34, 38) with minor modifications. Briefly, frozen muscle samples were homogenized in protein extraction buffer (10 mmol/L sodium phosphate and 2% SDS, pH = 7.0) containing protease inhibitor (Halt protease inhibitor cocktail, Thermo Fisher Scientific, Inc.) on ice, centrifuged for 15 min at 10,621 *g* at 4°C, and the resultant supernatant collected. Protein concentrations were determined colorimetrically using the Pierce bicinchoninic acid (BCA) Protein Assay (Thermo Fisher Scientific, Inc.). Samples were loaded onto precast 4–20% polyacrylamide gels (Bio-Rad, Hercules, CA), separated by molecular weight, and transferred onto nitrocellulose membranes. To confirm equal sample loading, all membranes were stained with Ponceau S stain (PonS) and imaged using the Azure Biosystems c600 imaging system. In all instances, total-lane optical density was similar for all groups indicating successful loading. Membranes were washed in Tris-buffered saline-Tween 20 (TBST) (50 mmol/L Tris·HCl, 150 mmol/L NaCl, 0.1% Tween 20, pH 7.4) to remove the PonS, blocked in 5% nonfat, dehydrated milk dissolved in TBST for 1 h, washed with TBST, and then incubated in primary antibody at 4°C overnight. Primary and secondary antibodies were diluted in TBST, unless otherwise noted, as follows: 4-hydroxynonenal (4-HNE) (Abcam, ab48506, primary 1:1,000 1% milk, secondary 1:2,000 2.5% dehydrated milk solution), calsequestrin [Santa Cruz Biotechnology (SCB), primary 1:500, secondary 1:1,000], CamKII (SCB, sc-5306, primary 1:500, secondary 1:1,000), catalase [Cell Signaling Technology (CST), No. 14097, primary 1:1,000, secondary 1:2,000], Cu/Zn superoxide dismutase (SOD1) (Abcam, ab13498, primary 1:1,000, secondary 1:2,000), glutathione peroxidase 1 (GPX1) (Abcam, ab22604, primary 1:1,000, secondary 1:2,000), malondialdehyde (MDA) (Abcam, ab27642, primary 1:1,000, secondary 1:2,000), Mn-superoxide dismutase (SOD2) (Abcam, ab13533, primary 1:1,000 milk, secondary 1:2,000), phospholamban (PLB) (Novus Biologicals, No. BP2-19807, primary 1:3,000 5% milk, secondary 1:3,000 5% milk), plasma membrane calcium ATPase (PMCA) (SCB, sc-271917, primary 1:500, secondary 1:1,000), sarcoendoplasmic calcium ATPase (SERCA2a) (CST, No. 9580, primary 1:1,000, secondary 1:2,000).

After overnight incubation, membranes were washed in TBST, rocked in secondary antibody for 1 h at room temperature, and washed again in TBST. Membranes were then rocked in enhanced chemiluminescence (Bio-Rad, Hercules, CA), and the resultant protein bands were detected using the Azure Biosystems c600 imaging system. The captured bands were quantified using the AzureSpot Software with automated band detection.

### Oxidative Stress

8-Isoprostane is produced by oxidation of phospholipids by reactive oxygen species. The 8-isoprostane competitive ELISA was used to measure free 8-isoprostane within samples (Cayman Cat. No. 516351). Approximately 50 mg frozen muscle tissue was homogenized in extraction buffer (50 mM sodium phosphate, 1 mM EDTA, pH = 7.0) to assess 8-isoprostane concentration. This assay is a competitive ELISA between 8-isoprostane and an 8-isoprostane-acetylcholinesterase (AChe) conjugate. The reaction between the AChe and Ellman’s reagent produces a color and absorbs at 420 nm. The amount of color produced is inversely proportional to the amount of free 8-isoprostane present (expressed in pg/mL).

Protein carbonyl concentration was determined spectrophotometrically using the Cayman Protein Carbonyl Colorimetric Assay Kit (Cat. No. 10005020). Briefly, ∼50 mg of frozen muscle samples were homogenized in phosphate buffer (50 mM sodium phosphate, 1 mM EDTA, pH = 7.0), centrifuged for 15 min at 10,000 *g* at 4°C, supernatant collected, and protein concentration determined. All samples were normalized to a protein concentration of 2.7 mg/mL, and the assay was preformed per manufacturer’s instructions. Protein carbonyl content is expressed in nmol/mg.

To assess DNA damage, DNA was isolated from ∼20 mg of powdered muscle tissue using the DNeasy blood and tissue kit (Qiagen, Cat. No. 69504) according to manufacturer’s instructions. DNA concentration and purity were determined using a Nanodrop Spectrophotometer and quality assured by 260/280 nm ratio. DNA damage was assessed using a qPCR-based method as previously described (39). The assay is dependent on a structural modification to the template DNA, which limits restriction enzyme (RE) digestion. Reaction mixes were made for the Taq1 restriction digest and undigested controls. The Taq1 reaction mix contained 10 µL iTaq Univer SYBR Green master mix (Bio-Rad), 2 µL forward and 2 µL reverse primers at a concentration of 4 mM, 1 µL Taq1 RE, and 3 µL water, for a total volume of 20 µL. Reaction mix for the undigested control contained the same components without the addition of the Taq1, and water volume was adjusted accordingly. Target primer sequences for a mitochondrial and nuclear gene are provided in Table 1. The cycling parameters were as follows: 95°C for 5 min, 15 s at 95°C for denaturation, and 30 s at 60°C for annealing and elongation for 40 cycles. Melting curves were inspected to confirm amplification of a single product. DNA damage frequency was calculated as 2^−(CT Taq1 – CT undigested)^.

**Table 1.** qPCR primer sequences

Target Gene	Forward Primer	Reverse Primer
*ND1*	CACAACACAAGAGCACATTTG	CGTGGGTATGATGCTCGGATTCA
*Ndufa9*	CCTGCTTCTGCAGGTGTTTAC	GTTATGCATTGAGTGTCTGAAGGCC

We also assessed DNA damage by measuring 8-hydroxy-2′-deoxyguanosine (8-OHdG) via ELISA (Cayman Cat. No. 589320) according to the manufacturer’s instructions. Briefly, DNA was diluted in nuclease-free water and digested with Nuclease P1 and alkaline phosphatase to a final concentration of 6 ng/µL before preforming the assay. Absorbance was read at 420 nm and DNA damage was expressed in pg/mL.

### Enzymatic Activities

Catalase activity (nmol/min/mL) was measured using a Catalase Assay Kit (Cayman Chemical, Cat. No. 707002) according to the manufacturer’s instructions. Briefly, 25–50 mg frozen tissue was homogenized in phosphate buffer (50 mM sodium phosphate, 1 mM EDTA, pH 7.0). The formation of formaldehyde from the reaction of catalase and methanol in the presence of hydrogen peroxide was measured spectrophotometrically at 540 nm.

Glutathione peroxidase (GPX) activity was measured according to the manufacturer’s instructions (Cayman, Cat. No. 703102). Approximately 30 mg of frozen tissue was homogenized in Tris·HCl buffer (50 mM Tris·HCl, 5 mM EDTA, 1 mM DTT, pH 7.5), and all samples were diluted to 4 µg/µL. The rate of reduced absorbance at 340 nm is directly proportional to sample GPX activity (expressed as μmol/min/mL).

Total SOD activity was measured colorimetrically from 30 mg of muscle tissue homogenized in HEPES buffer (20 mM HEPES buffer, 1 mM EGTA, 210 mM mannitol, 70 mM sucrose, pH = 7.2) (Cayman Cat. No. 706002). Absorbance at 440 nm was determined using a microplate reader (Bio-Tek, Winooski, VT). Total SOD activity was expressed as U/mL/mg of protein.

Abundance of reduced (GSH) and oxidized glutathione (GSSG) were measured via targeted metabolomic analysis. Tissue preparation and a detailed protocol have been previously described (40, 41). Briefly, frozen skeletal muscle samples were lyophilized overnight before bead mill homogenization in ice-cold methanol:acetonitrile:water extraction buffer containing a mixture of internal standards. Homogenates were centrifuged for 10 min at 21,000 *g*, and the resultant metabolite extracts were transferred to autosampler vials and dried. Dried metabolite extracts were reconstituted in methoxyamine (MOX) in anhydrous pyridine, vortexed for 5 min, and heated for 1 h at 60°C. *N*,*O*-bis(trimethylsilyl)trifluoroacetamide (TMS) was added to each sample. Samples were analyzed on a Thermo Q Exactive hybrid quadrupole Orbitrap mass spectrometer. Acquired LC-MS data were processed by Thermo Scientific TraceFinder.1 software, and metabolites were identified based on the University of Iowa Metabolomics Core facility standard-confirmed, inhouse library. NOREVA was used for signal drift correction (42). Data were normalized to total ion signals and MetaboAnalyst 4.0 was used for further statistical processing and visualization (43).

### Calcium ATPase Activity

Ca^2+^ ATPase activity (nmol/min/mL) was measured colorimetrically through inorganic phosphorus production using a Ca^2+^-ATPase Activity Assay Kit (Elabscience, Cat. No. E-BC-K212-M) according to manufacturer’s instructions. Briefly, ∼30 mg frozen tissue was homogenized in normal saline (0.9% NaCl) centrifuged for 15 min at 10,000 *g*, supernatant collected, and protein concentration determined. The optical density was measured at 660 nm using a microplate reader. Ca^2+^ ATPase activity was expressed as U/g of protein.

### Histological Analysis

The oxidative portion of the semitendinosus was fixed in 4% paraformaldehyde overnight and stored in 70% EtOH. Fixed tissues were embedded in paraffin, cut in 4-µm sections, and stained with hematoxylin and eosin (H&E) or Alizarin red (ARS). Before staining, H&E slides were deparaffinized in xylene, whereas ARS slides were deparaffinized in xylenes (Fisher Scientific, Cat. No. AC422685000). Both sets of slides were then rehydrated in EtOH and then rinsed in running water for 1 min. H&E staining was performed according to standard techniques and were dehydrated and cleared in a series of EtOH and xylene washes. For ARS, after slides were deparaffinized and rehydrated, slides were exposed to 2% Alizarin red (pH 4.16) for 2 min, dipped 20 times in acetone, dipped 20 times in a 1:1 acetone-xylene solution, and rinsed in three consecutive 1-min baths of xylenes. All slides were air-dried and mounted with cover slips using Permount Mounting Medium and sealed with nail polish. For H&E images, the entire muscle cross-section on the slide was subjectively evaluated by a boarded veterinary anatomic pathologist under blinded conditions. For ARS, five random images at ×20 magnification were taken for each section under blinded conditions. All images were objectively measured with OpenLab (Improvision) to quantify areas of Ca^2+^ deposition using density slicing and are expressed as a percentage of the total cross-sectional area.

### Statistical Analysis

Sample size was estimated using differences in biochemical outcomes in our previous work utilizing a porcine HS model (22, 34, 38). Normality was assessed in all measures using the Shapiro–Wilk test. Data that were not normally distributed were log transformed before statistical testing. All data were analyzed using the proc MIXED procedure in SAS version 9.4 (SAS Inst. Inc., Cary, NC). The model consisted of main effects of environment, sex, and an environment × sex interaction. Preplanned contrasts were utilized to evaluate the effects of environment within a sex (i.e., TN male vs. HS1 male) and sex within an environment (i.e., HS1 male vs. HS1 female). Data are presented in figures as least square means ± SE. Statistical significance was considered as *P* < 0.05.

## RESULTS

A detailed analysis of the physiological response to this heat intervention has been previously reported (35). Briefly, heat exposure elevated rectal temperature in males (40.4 vs. 39.4°C) and females (40.3 vs. 39.2°C) following 1 day of HS. After 7 days of HS, pigs of both sexes had elevated rectal temperature compared with pre-HS values (males: 40.4 vs. 39.4°C; females: 40.0 vs. 39.2°C); however, rectal temperature from females was decreased compared with HS7 males (*P* < 0.05; 0.4°C) and compared with HS1 females (*P* < 0.05; 0.3°C).

**Figure 1.**
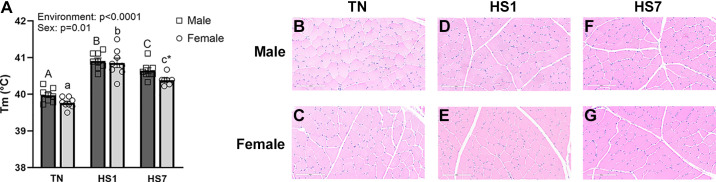
Heat stress (HS) differentially increases muscle temperature (*T*_m_) but does not induce histopathological changes in males and females. *A*: muscle temperature increased following 1 (HS1) and 7 days of HS (HS7); however, *T*_m_ was reduced by 7 days compared with 1 day, and females were lower than males at HS7. *B*–*G*: hematoxylin and eosin (H&E) representative images from males and female oxidative skeletal muscle. Data are shown as least square means ± SE (*n* = 7–8/group). Significant main effects are indicated as text. Significant differences between male groups [thermoneutral (TN)M, HS1M, HS7M] are indicated by different uppercase letters (*P* < 0.05). Significant differences between female groups (TNF, HS1F, HS7F) are indicated by different lowercase letters (*P* < 0.05). *Significant difference between males and females within an environmental treatment (*P* < 0.05).

Environment-induced HS increased *T*_m_ compared with TN (*P* < 0.0001), and males had higher *T*_m_ (*P* < 0.01) compared with females (Fig. 1*A*). Specifically, HS1 and HS7 male muscle temperature was 40.9°C and 40.6°C, whereas TN males were 40.0°C (*P* < 0.01). Similarly, skeletal muscle from HS1 and HS7 females was 40.8°C and 40.4°C, and TN females had a muscle temperature of 39.8°C (*P* < 0.01). Muscle temperature between HS1 males and females was similar; however, *T*_m_ was lower following 7 days of HS compared with 1 day (*P* < 0.05) in both males and females. Finally, after 7 days of HS, *T*_m_ was 0.25°C greater in males than females (*P* < 0.05; Fig. 1*A*). Despite elevations in muscle temperature caused by HS, muscle histopathological injury following H&E staining was not apparent (Fig. 1, *B*–*G*).

**Figure 2.**
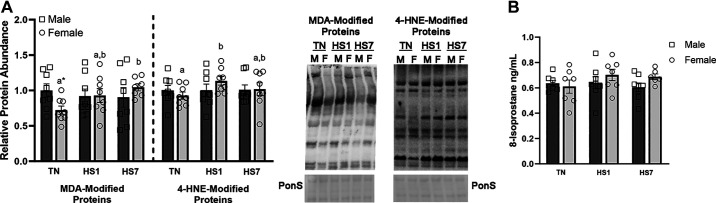
Assessment of lipid oxidation in oxidative skeletal muscle from males and females following 1 (HS1) and 7 (HS7) days of heat stress (HS). *A*: relative protein abundance of malondialdehyde (MDA) and 4-hydroxynonenol (4-HNE) modified proteins was measured via Western blot analysis. Ponceau S stain (PonS) was used as a loading control. *B*: 8-isoprostane concentration was determined using a competitive ELISA (expressed as ng/mL). Values represent the least square means ± SE (*n* = 8/group). Significant main effects are indicated as text. Significant differences between female groups [thermoneutral (TN)F, HS1F, HS7F] are indicated by different lowercase letters (*P* < 0.05). *Significant difference between males and females within an environmental treatment (*P* < 0.05).

**Figure 3.**
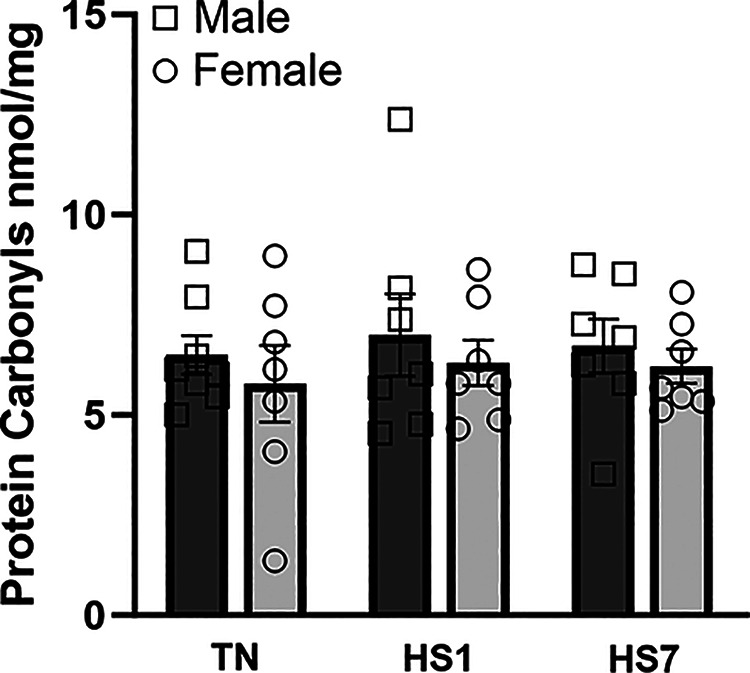
Heat stress (HS) does not increase protein carbonylation in male and female oxidative skeletal muscle. Protein carbonyls were assessed colorimetrically (expressed as nmol/mg). Data are shown as least square means ± SE (*n* = 7/group). HS1, 1 day of HS; HS7, 7 days of HS; TN, thermoneutral.

To assess oxidative stress, we quantified markers of oxidative damage to lipids, proteins, and nucleic acids. Relative protein abundance of MDA- and 4-HNE-modified proteins did not differ by main effects of sex, environment, or the interaction (Fig. 2*A*). Heat-stressed males remained similar to TN males for both MDA- and 4-HNE-modified proteins. However, preplanned contrasts showed relative MDA-modified proteins were increased by 45% in HS7 females compared with TN females (*P* = 0.02). In addition, HS1 females had elevated 4-HNE modified proteins compared with TN females (22%; *P* = 0.05). Free 8-isoprostane (Fig. 2*C*) and protein carbonyls (Fig. 3) were similar between groups.

**Figure 4.**
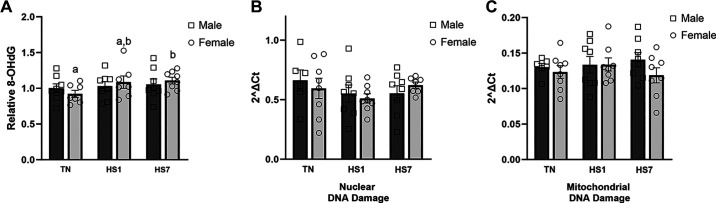
Assessment of oxidative skeletal muscle DNA oxidative modification following 1 (HS1) and 7 days (HS7) of heat stress (HS) in males and females. *A*: relative 8-hydroxy-2'-deoxyguanisine (8-OHdG) concentration was measured via competitive ELISA. qPCR-based approach to assess nuclear (*B*) and mitochondrial oxidative (*C*) damage following 1 and 7 days of HS. Values represent the least square means ± SE (*n* = 6–8/group). Significant differences between female groups [thermoneutral (TN)F, HS1F, HS7F] are indicated by different lowercase letters (*P* < 0.05).

Mitochondrial and nuclear DNA damage was assessed following HS in males and females. Relative 8-OHdG concentration remained similar as a main effect of environment, sex, and the interaction; however, HS7 animals had a higher relative abundance of 8-OHdG than TN (12%; *P* = 0.05; Fig. 4*A*), which was primarily driven by the HS7 females as they were increased compared with TN females (20%; *P* = 0.05). With the use of a qPCR-based approach, neither nuclear nor mitochondrial DNA damage was apparent (Fig. 4, *B* and *C*).

**Figure 5.**
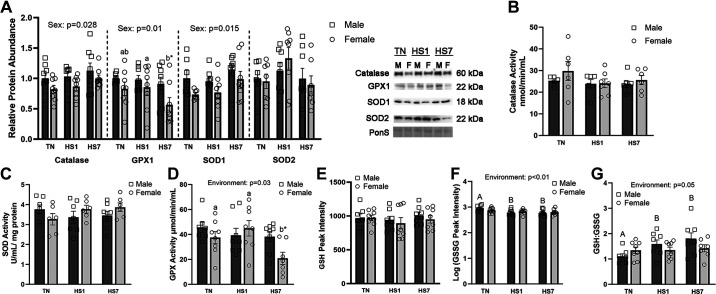
Assessment of antioxidant enzymes following 1 (HS1) and 7 days (HS7) of heat stress (HS) in oxidative skeletal muscle from males and females. *A*: relative protein abundance of antioxidant enzymes following HS. Ponceau S stain (PonS) was used as a loading control. Enzymatic activity of catalase (nmol/min/mL) (*B*), total superoxide dismutase (SOD; U/mL) (*C*), and glutathione peroxidase (GPX; nmol/min/mL) (*D*) were measured. Peak intensity of reduced (GSH) (*E*) and oxidized glutathione (GSSG) (*F*) and the ratio of GSH:GSSG (*G*) are shown. Oxidized glutathione was not normally distributed and the data were log transformed before statistical analysis. A main effect of environment (*P* < 0.05) and/or sex (*P* < 0.05) is indicated. Values represent the least square means ± SE (*n* = 6–8/group). Significant main effects are indicated as text. Significant differences between male groups [thermoneutral (TN)M, HS1M, HS7M] are indicated by different uppercase letters (*P* < 0.05). Significant differences between female groups (TNF, HS1F, HS7F) are indicated by different lowercase letters (*P* < 0.05).

As antioxidants are crucial in maintaining and restoring redox balance, we measured protein abundance and activity of several antioxidants (Fig. 5*A*). There was a main effect of sex on catalase (*P* = 0.03), GPX1 (*P* = 0.01), and SOD1 (*P* = 0.01) abundance such that males had 17–28% more than females, but relative protein expression of antioxidant enzymes did not change as a main effect of environment or the environment × sex interaction. We observed decreased GPX1 protein abundance between HS7 females compared with HS1 females (*P* = 0.05), whereas male groups remained similar between groups. In addition, HS7 female GPX1 protein abundance was reduced 38% compared with HS7 males (*P* = 0.02).

Enzymatic activities of catalase and SOD were similar between groups (Fig. 5, *B* and *C*). GPX activity had a main effect of environment (*P* = 0.03; Fig. 5*D*), which was driven primarily by the HS7 female group; however, there was no effect of sex or the environment × sex interaction. By 7 days of HS in females, GPX activity was reduced compared with TN females (44%; *P* = 0.03), HS1 females (54%; *P* = 0.02), and HS7 males (45%; *P* = 0.02), whereas all male groups remained similar.

Reduced glutathione (GSH) was not altered by environment or biological sex (Fig. 5*E*). Oxidized glutathione (GSSG) data were log transformed to achieve normal distribution and was reduced as a main effect of environment (*P* < 0.01; Fig. 5*F*). The reduction was driven by the HS males as HS1 and HS7 males were decreased compared with TN males (HS1-5%; *P* < 0.05; HS7-6%; *P* < 0.05), whereas females remained similar between groups. The GSH:GSSG ratio was increased as a main effect of environment (*P* = 0.05) driven by the HS males. Specifically, HS1 and HS7 males were increased 44% and 63% compared with TN males, respectively (*P* = 0.03; *P* < 0.01), whereas HS females remained similar to TN females (Fig. 5*G*).

**Figure 6.**
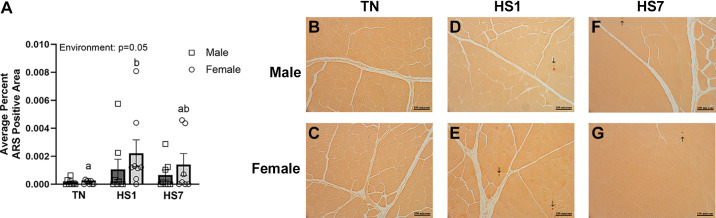
Heat stress increases calcium deposition in oxidative skeletal muscles of females. *A*: average percent alizarin red stain (ARS)-positive area was increased following 1 (HS1) day of heat stress (HS) in female but not male oxidative skeletal muscle. *B*–*G*: representative images from ARS stain. Values represent the least square means ± SE (*n* = 7–8/group). Significant differences between female groups [thermoneutral (TN)F, HS1F, HS7F] are indicated by different lowercase letters (*P* < 0.05). Areas of calcium deposition are marked with black arrow (↑).

Given the role Ca^2+^ homeostasis plays in oxidative stress and overall muscle health, we assessed Ca^2+^ deposition via histological staining. We discovered an increase in Ca^2+^ deposition in heat-stressed skeletal muscle compared with TN (*P* = 0.05; Fig. 6, *A*–*G*). Specifically, HS1 females had greater skeletal muscle Ca^2+^ deposition than TN females (*P* = 0.02), HS7 females remained similar to TN and HS1 females, whereas Ca^2+^ deposition in males remained similar between all groups.

**Figure 7.**
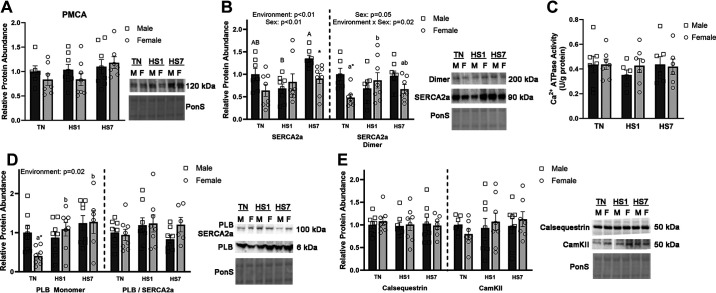
Calcium regulatory proteins were assessed following 1 (HS1) and 7 days (HS7) of heat stress (HS) in oxidative skeletal muscle from males and females. Relative protein abundance of Ca^2+^ pumps plasma membrane calcium ATPase (PMCA) (*A*) and sarco(endo)plasmic reticulum Ca^2+^ ATPase (SERCA)2a (*B*) full-length protein and dimer were assessed. *C*: Ca^2+^ ATPase activity was measured (expressed as U/g protein). *D*: phospholamban (PLB), a SERCA2 inhibitor, was assessed in its monomeric and SERCA2-bound form. *E*: Ca^2+^ handling proteins calsequestrin and CamKII were also measured. Ponceau S stain (PonS) was used as a loading control. Values represent the least square means ± SE (*n* = 7–8/group). Significant main effects are indicated as text. Significant differences between male groups [thermoneutral (TN)M, HS1M, HS7M] are indicated by different uppercase letters (*P* < 0.05). Significant differences between female groups (TNF, HS1F, HS7F) are indicated by different lowercase letters (*P* < 0.05). *Significant difference between males and females within an environmental treatment (*P* < 0.05).

Given the alterations in Ca^2+^ homeostasis, we examined the Ca^2+^ pumps, PMCA, and SERCA2a, and the Ca^2+^ handling proteins, calsequestrin, and CamKII. PMCA did not have a main effect of environment, sex, or the interaction (Fig. 7*A*). There was a main effect of environment (*P* < 0.01) and sex (*P* < 0.01) on SERCA2a protein abundance, such that abundance was increased following HS and males had more SERCA2a relative protein abundance than females (Fig. 7*B*). In addition, relative abundance of SERCA2a dimer, indicative of SERCA2a oxidative modification (44), had a main effect of sex (*P* = 0.05) such that males had greater relative protein abundance than females. There was also an environment × sex interaction (*P* = 0.02) as females had increased SERCA2a dimer protein abundance following HS, whereas males remained similar between groups (Fig. 7*B*). Despite alterations in protein abundance in PMCA and SERCA2a, Ca^2+^ ATPase activity remained similar between groups (Fig. 7*C*). Phospholamban (PLB), a SERCA2a inhibitor, exists in several forms. The PLB monomer was increased as a main effect of environment (*P* = 0.02). The main effect was primarily driven by HS females as both HS1 and HS7 females were elevated compared with TN (*P* = 0.02 and *P* < 0.01, respectively; Fig. 7*D*). Of note, TN males had more PLB relative protein abundance than TN females (*P* = 0.02). Furthermore, PLB bound to SERCA2a (45) was neither impacted by environment nor sex (Fig. 7*D*). Calsequestrin and CamKII remained similar between groups (Fig. 7*E*).

## DISCUSSION

Prolonged exposure to high environmental temperatures can result in a multisystemic stress that threatens human health and efficient agricultural production (10, 46). Oxidative stress is commonly induced by HS in skeletal muscle (15, 18, 47, 48), as well as other tissues (49–51). Recent data from our laboratory suggest that females may be more susceptible to skeletal muscle oxidative stress than males during HS (34); however, the extent to which oxidative stress differs between males and females following HS remains largely unknown. In the study presented herein, we discovered that skeletal muscle from females may be more susceptible to oxidative stress than skeletal muscle from males following HS in a porcine HS model. Furthermore, this may be due, at least in part, to compromised Ca^2+^ homeostatic control and reduced antioxidant capacity.

Pathologies such as muscle atrophy, cardiac ischemia-reperfusion, type II diabetes, and Duchenne muscular dystrophy (DMD) share Ca^2+^ dysregulation as a common feature, which has been causatively linked to impaired mitochondrial function and oxidative stress (24, 52). Consistent with this notion, we observed increased skeletal muscle Ca^2+^ deposits in heat-stressed skeletal muscle, particularly in females. Although clearly increased, the extent of calcium deposition following HS is mild, particularly when compared with muscle pathologies such as DMD (53). Nevertheless, these data raise the exciting possibility that Ca^2+^ dysregulation is a mediator of HS-induced oxidative stress. Our working model of skeletal muscle HS-mediated pathology is initiated by a loss of Ca^2+^ control, followed by an excess of cytosolic and mitochondrial Ca^2+^, and subsequent slowing and uncoupling of the electron transport chain (ETC). Using these tissues, we previously discovered that mitochondrial function was more negatively impacted by HS in muscle from females than from males and that HS caused sex-specific alterations of ETC complex function, as females relied more on complex I and males had a greater reliance on complex II (21). When mitochondrial membrane potential is altered, ostensibly by excess Ca^2+^, complex I is more susceptible to *e*^−^ leak and superoxide production. Consistent with this notion, markers of oxidative stress were only increased in skeletal muscle from females.

The Ca^2+^ pump, SERCA2a, moves Ca^2+^ from the cytoplasm into the sarcoplasmic reticulum (SR). Calcium-induced Ca^2+^-ATPase activity and maximal Ca^2+^ ATPase activity have been shown to decrease following HS, in vitro, at temperatures of ∼40–41°C compared with 37°C (30, 54). In the current experiment, muscle temperature following HS was ∼40.4–40.9°C, which we note is suspiciously close to the aforementioned temperature that caused impaired function, raising the possibility of decreased SERCA2a function in muscles considered herein. In addition to thermal denaturation, SERCA2a appears to be particularly susceptible to oxidative modification, which alters pump capacity and performance (32). Oxidative modification of SERCA2a can result in protein dimerization (44), which was increased following HS in muscle from females, but not males, and is consistent with our findings of increased oxidative stress in muscles from females. Despite the changes in skeletal muscle temperature and oxidative injury, total Ca^2+^ ATPase activity was similar between groups. Although this was surprising, as we noted SERCA2a protein abundance was increased following 7 days of HS in muscle from both sexes, it is reasonable to suggest this elevation in protein abundance is a compensatory mechanism to maintain SERCA activity under HS conditions. Indeed, such a change may reflect cellular underpinnings of acclimation. Moreover, while a detailed exploration of the SERCA interactome was beyond the scope of this investigation, aside from phospholamban, another intriguing possibility is maintenance of SERCA activity via interaction with sarcolipin and/or myoregulin, among other proteins (54). We are not aware of previous work exploring the role of SERCA during a hyperthermic stress; however, SERCA activity appears to be increased during hypothermia and is an important contributor to nonshivering thermogenesis (55–57). Ultimately, preservation of Ca^2+^ ATPase activity discovered herein makes SERCA2a dysregulation an unlikely mechanism driving Ca^2+^ dysregulation, despite the potential for thermal denaturation and oxidative modification. This leads us to suspect that proteins associated with Ca^2+^ release from the SR, such as ryanodine receptors, may be contributing to excess sarcoplasmic Ca^2+^ during HS.

Slowing of the ETC drives electron leakage, and subsequent production of reactive oxygen species (ROS), which can modify lipids, DNA, and proteins. Of the macromolecules, lipids, particularly polyunsaturated fatty acids, are especially susceptible to free radical attack and oxidative modification (58). Consistent with previous findings from our laboratory (15, 34), in this investigation, 4-HNE- and MDA-modified proteins increased with 1 and 7 days of HS, respectively, in muscle from females, but not males. Furthermore, 8-OHdG is a marker of oxidative modification to DNA (59) and has been previously reported to be increased in skeletal muscle following HS (20). In this investigation, we discovered increased relative 8-OHdG concentration between TN and HS females, whereas males remained similar between groups.

To better understand the preservation of redox balance in muscle from males and a shift to a pro-oxidant state in skeletal muscle from females, we probed several antioxidants. Superoxide dismutase converts superoxide into hydrogen peroxide (H_2_O_2_), and catalase and GPX reduce H_2_O_2_ to H_2_O. Furthermore, GPX also reduces lipid hydroperoxides through the conversion of GSH to GSSG. An imbalance in antioxidant activity between SODs and catalase and/or GPX may promote oxidative stress via increased cellular H_2_O_2_ (60, 61). Indeed, in females, while SOD activity is maintained with HS, the approximate 50% reduction in GPX activity may contribute to a pro-oxidant intracellular environment in muscle from females.

Glutathione is a nonprotein thiol and cellular redox regulator, and the ratio of GSH:GSSG is frequently used as a marker of redox status (62–64). In this investigation, HS caused a reduction in GSSG in males, which led to an increased GSH:GSSG ratio, and is associated with maintenance of redox balance (65). Although the elevation in GSH:GSSG was driven by lowered GSSG rather than increased GSH, oxidative stress was not apparent in HS males, consistent with an elevated ratio (66–68). The mechanism underlying the reduction in GSSG in HS males remains unknown; however, we speculate the maintenance of GSH levels is due, in part, to glutathione reductase activity, which converts GSSG back to GSH. This ratio may be further supported by alterations in purine metabolism (69, 70); however, the interrogation thereof was beyond the scope of this investigation.

### Perspectives and Significance

With global temperatures and the frequency of severe heat events increasing, heat stress is a current and growing threat to human health and efficient animal production worldwide. It has been well established that HS induces oxidative stress in a variety of tissues, including skeletal muscle. The data presented herein indicate that biological sex is a key mediator of HS-mediated changes as we discovered evidence supporting a role of Ca^2+^ dysregulation in heat-stressed muscles was more notable in muscle from females than from males. Moreover, muscle from females had more oxidative injury than muscle from males, even though muscles from females were cooler than males. These data raise the possibility that biological sex plays an underlying role in pathologies caused by a changing environment and should be considered in the development and application of countermeasures in the context of human medicine and agricultural production. To better appreciate the impact of biological sex on these outcomes we are eager to assess skeletal muscle from intact males and in females at various stages of the estrus cycle in future investigations.

## DATA AVAILABILITY

The data that support the study findings are available upon request.

## GRANTS

This work was supported in part by US Department of Agriculture Grants 2019-07859 (to J.T.S) and 2022-67011-36636 (to T.E.R).

## DISCLOSURES

No conflicts of interest, financial or otherwise, are declared by the authors.

## AUTHOR CONTRIBUTIONS

R.P.R., S.H.W.-S., L.H.B., and J.T.S. conceived and designed research; T.E.R., M.R., A.D.F., S.Q.E.Y., A.M., L.H.B., and J.T.S. performed experiments; T.E.R., S.Q.E.Y., A.M., and J.T.S. analyzed data; T.E.R., A.M., and J.T.S. interpreted results of experiments; T.E.R. prepared figures; T.E.R. and J.T.S. drafted manuscript; T.E.R., M.R., A.D.F., S.Q.E.Y., A.M., R.P.R., S.H.W.-S., L.H.B., and J.T.S. edited and revised manuscript; T.E.R., M.R., A.D.F., S.Q.E.Y., A.M., R.P.R., S.H.W.-S., L.H.B., and J.T.S. approved final version of manuscript.
